# Adolescents' Longitudinal School Engagement and Burnout Before and During COVID‐19—The Role of Socio‐Emotional Skills

**DOI:** 10.1111/jora.12654

**Published:** 2021-08-26

**Authors:** Katariina Salmela‐Aro, Katja Upadyaya, Janica Vinni‐Laakso, Lauri Hietajärvi

**Affiliations:** ^1^ University of Helsinki

**Keywords:** COVID‐19, school engagement and burnout, latent profiles, socio‐emotional skills, longitudinal

## Abstract

This longitudinal study examined school engagement and burnout profiles among early and middle adolescents before and during COVID‐19, and within‐class latent change and stability in students’ socio‐emotional skills the profiles. The longitudinal data were collected in fall 2019 and 2020 from 1381 5th to 6th, and 1374 7th to 8th grade students. Using repeated measures latent profile analyses based on school engagement and burnout we identified five study well‐being change profiles in both samples showing structural similarity: normative (53% sample 1; 69% sample 2), moderate‐decreasing (4%; 5%), high‐decreasing (17%; 10%), low‐increasing (6%;7%) and moderate‐increasing (20%; 10%) groups. The groups with increasing study well‐being showed simultaneous increase in intrapersonal socio‐emotional competencies but showed less changes in interpersonal outcomes.

The COVID‐19 pandemic has influenced the education sector all over the world and affected the learning of 1.6 billion children and young people in 200 countries (UNESCO, 2020). As a consequence, lockdowns, restrictions on movement, disruption of routines, physical distancing, curtailment of social interactions and deprivation of traditional learning methods have led to increased stress, anxiety, and mental health concerns among learners worldwide (UNESCO, 2020). Recent results have shown that especially middle and high school students experienced high levels of anxiety during the COVID‐19 pandemic (Hoyt, Cohen, Dull, Castro, & Yazdani, 2021; Styck, Malecki, Ogg, & Demaray, 2020). On the whole, COVID‐19 and its containment measures have created unique challenges for academic and psychological well‐being. To counteract negative developmental outcomes, resources must be identified that foster resilience in times of crisis. Therefore, the present research seeks to identify longitudinal change patterns in study‐related well‐being (e.g., engagement, burnout) before and during COVID‐19, and examine the simultaneous development of key socio‐emotional skills (e.g., resources) from the OECD socio‐emotional skills framework (Kankaras & Suarez‐Alvarez, 2019) including curiosity, grit, social engagement, belongingness and academic buoyancy (Martin & Marsh, 2008) that support study‐related well‐being.

School engagement and burnout have taken a prominent place in recent developmental psychological and educational research because they provide a good overview of students’ academic and psychological functioning (Salmela‐Aro & Upadyaya, 2014), and because of their potential for predicting poor academic achievement, student misbehavior and school dropout (Li & Lerner, 2011; Wang & Peck, 2013). Both school engagement and burnout have been widely used across different age samples, and educational contexts (Tuominen‐Soini & Salmela‐Aro, 2014). However, this study is one of the first to examine longitudinally the changes in both school engagement and burnout before and during the COVID‐19 pandemic among early and middle adolescents.

Study‐related well‐being as a new research topic, often approached through school burnout and engagement, has quickly gained international attention which speaks to its perceived relevance across several nations (e.g., May, Bauer, & Fincham, 2015; Yang & Chen, 2016). School engagement can be defined as vigor, dedication, and absorption toward school (Salmela‐Aro & Upadyaya, 2012). School burnout, in turn, can be defined as a school‐related syndrome including exhaustion, negative cynical attitude toward school and feelings of inadequacy as a student (Salmela‐Aro, Kiuru, Leskinen, & Nurmi, 2009). School burnout can lead to depressive symptoms and increases the risk of dropping out from school four times more, whereas school engagement can promote both life satisfaction and success in future educational transitions (Bask & Salmela‐Aro, 2013; Salmela‐Aro & Upadyaya, 2014).

The number of studies on adolescents’ study‐related well‐being has been increasing in the recent years. However, only few studies have adopted person‐oriented approaches to describe subgroups of students with different burnout symptoms, also taking into account the positive side of school engagement (Virtanen, Lerkkanen, Poikkeus, & Kuorelahti, 2018). The advantage of person‐oriented approaches, in contrast to variable‐oriented approaches, is that they provide a method to capture individual differences and identify homogeneous subgroups in the target variables beyond overall mean level. Specifically, in this study we examined change profiles of students’ engagement and burnout, and identified different subpopulations of students experiencing varying levels of engagement and burnout before and during the COVID‐19 pandemic. Previous studies employing person‐oriented approaches have shown that while some highly engaged students ‘flourish’, others may simultaneously experience high engagement and burnout symptoms (Salmela‐Aro, Muotka, Alho, Hakkarainen, & Lonka, 2016; Virtanen, Lerkkanen, Poikkeus, & Kuorelahti, 2018). Moreover, socio‐emotional skills may protect students from burning out in their studies and promote their engagement (Salmela‐Aro & Upadyaya, 2020) even during global pandemic. The present study provides a unique contribution to the field by examining both 6th (early adolescents) and 8th (middle adolescents) grade students’ longitudinal engagement and burnout change profiles in association with socio‐emotional skills during the COVID‐19 pandemic.

Previous longitudinal studies have also shown that in adolescence school burnout slightly increases during secondary education and across the transition to secondary education, reflecting decreases in emotional school engagement (Salmela‐Aro, Read, Minkinen, Kinnunen, & Rimpelä, 2018; Wang, Chow, Hofkens, & Salmela‐Aro, 2015). School burnout increases especially among girls, however, immigrant boys experience higher levels of cynicism (Salmela‐Aro et al., 2018). Increases in burnout are often associated with decreases in engagement and may manifest as excessive internet use and increased depressive symptoms (Salmela‐Aro, Upadyaya, Hakkarainen, Lonka, & Alho, 2017).

Understanding the opportunities and resources that support students’ engagement preventing stress and burnout, as well as the demands that hinder engagement and promote burnout has become a priority for educational policy and practice (Wang & Eccles, 2012) and is particularly important in the time of global crisis. According to the demands‐resources model in school context (Salmela‐Aro & Upadyaya, 2014), the more demands (e.g., workload, study‐related stress) the students experience, the more school burnout they experience, whereas the resources, both school‐related and personal, such as socio‐emotional skills, are related to high level of school engagement and low level of school burnout (Salmela‐Aro & Upadyaya, 2014, 2020). Socio‐emotional competences are critical factors promoting students’ positive well‐being and development (Schoon, 2021). Students with adequate socio‐emotional skills might have the necessary resources to maintain a satisfactory school engagement without becoming burned out when facing the stressors caused by the fluctuations in study and social circumstances due to COVID‐19. In the present study the OECD socio‐emotional skills framework was used to identify the key socio‐emotional skills as predicting different school engagement‐burnout profile membership (Salmela‐Aro & Upadyaya, 2020). The OECD framework has identified five key socio‐emotional skills: (1) The ability to be self‐disciplined, persistent, and dedicate effort in achieving goals and completing tasks, such as *grit* (Duckworth, Peterson, Matthews, & Kelly, 2007), (2) the ability to control one’s emotional responses and moods, and to be positive and optimistic, such as *academic buoyancy*, (3) the ability to maintain positive relations and be sympathetic to others, such as *social engagement*, (4) the ability to engage with new ideas and generate novel ways to do or think, such as *curiosity*, and (5) the ability to engage with others, such as *belonginess* and *lack of loneliness*. High socio‐emotional skills are often associated with high engagement (Salmela‐Aro & Upadyaya, 2020). In turn, school burnout is approached as a mismatch between individual’s socio‐emotional skills, and demands imposed by the school context which cause students to experience depletion of energy without gaining appropriate returns. In line with the demands‐resources model, two processes can be identified: a motivational process, in which resources lead to increased engagement, and a health impairment process in which demands lead to strain and health problems (Bakker & Demerouti, 2006). In support of the model, findings from longitudinal research show that school engagement and burnout also spill over from the school domain‐specific context to general ill‐ and well‐being (Salmela‐Aro & Upadyaya, 2014), and to further achievements, aspirations, educational choices, and pathways (Bask & Salmela‐Aro, 2013; Upadyaya & Salmela‐Aro, 2015).

## The Onset of the Pandemic in Finland

In Finland, COVID‐19 pandemic has been the most severe in Helsinki municipality area, and it affected students at all school levels. During the outburst of COVID‐19 cases in spring 2020 compulsory education was rapidly switched to remote teaching. How schools in Helsinki area were equipped with digital skills and devices, and how remote teaching was organized varied. Depending on the teacher, classes were held online or students were working independently at least part of the time, however, social interaction with peers was often emphasized during the online lessons. In fall 2020, compulsory education was organized in schools applying various safety measures and restrictions administered by the Education Division of city of Helsinki. Students’ contacts with their peers outside their own classroom were limited and reduced. In the classrooms, students were seated with social distance, and collaboration with other students was limited.

## The Present Study

In the present study, we identify longitudinal latent profiles of school engagement and burnout among elementary and middle school students. We expect that the students who have good socio‐emotional skills are more likely to belong to the engaged profile (Salmela‐Aro & Upadyaya, 2020), and that COVID‐19 is likely to increase the risk of burnout and decrease school engagement both in early and middle adolescence in elementary and middle school. During the COVID‐19 pandemic students’ social relationships dramatically reduced, causing high stress among middle and high school students (Styck et al., 2020). We expect further that socio‐emotional skills can buffer the negative changes by COVID‐19. Academic buoyancy, defined as student’s ability to successfully deal with academic setbacks and challenges, for example, can buffer these negative effects (Martin & Marsh, 2008, Martin & Marsh, 2009). Recently, also the role of grit and curiosity have been suggested as important buffering variables from school burnout (Salmela‐Aro & Upadyaya, 2020; Tang, Guo, Wang, & Salmela‐Aro, 2019) which may promote engagement and academic success (Duckworth et al., 2007). In addition, social skills, such as lack of loneliness and social belongingness, are key factors supporting school engagement (Kankaras & Suarez‐Alvarez, 2019).

The first aim of the present longitudinal study was to examine what kinds of profiles of school engagement and burnout can be identified among early and middle adolescent students. We expect to identify at least four profiles: burned out, engaged, increasing burnout, and increasing engagement (H1). Further, we expect that during the COVID‐19 both size of the burned out and increasing burnout profiles would increase in both age groups, especially among the middle adolescents (H2). Second, we examine the extent to which these profiles differ in terms of socio‐emotional skills defined in the OECD framework as curiosity, grit, academic buoyancy, social engagement, belongingness, and loneliness. We expect that socio‐emotional skills protect from burnout (H3).

## METHOD

### Participants and Procedure

This study is a part of the ongoing Growing Mind (GM) and Bridging the Gaps (GAPS) Studies (2019–2023) in which two age cohorts participate in 2‐year longitudinal study in Helsinki metropolitan area. The data consists of 2755 students from two different age groups. Sample 1 consist of 1381 elementary students (40.7% females; 39.2% males, 2.1% non‐binary, missing 17.9%) followed from 5th to 6th grade, born in 2008, and sample 2 consist of middle school students 1374 (40.0% females; 37.9% male, 1.9% non‐binary, missing 20.2%) followed from 7th to 8th grade, born in 2006. The data was collected in fall semesters of 2019 and 2020 when students in sample 1 were at grade 5 to 6 (first year age *M* = 10.83, *SD* = 0.41, min 9–max 12), and in sample 2 were at grade 7 to 8 (first year age *M* = 12.82, SD = 0.42, min 11–max 16). The participants completed an one hour online questionnaire measuring school engagement, school burnout, socio‐emotional skills, and background variables. Participation was voluntary, and written active consent was obtained from all the student participants and their parents. The study protocol was pre‐examined and approved by the University of Helsinki Ethical Review Board in the Humanities and Social and Behavioural Sciences. Furthermore, the research plan was pre‐examined and approved by the Education Division of the city of Helsinki. Detailed information on the preliminary analyses conducted to examine the reliability and validity of the measures, as well as a detailed description of the substantive analysis process are presented in Supplementary File S1 (EITHER INCLUDED BY THE JOURNAL OR https://osf.io/s6rkq/?view_only=11672f256b754c4c9977a0b369e5c0a5).

### Measures

The first‐order correlations, mean and standard deviations of the variables are presented in Table 1A and 1B.

**Table 1:** First‐Order Correlations, Means and Standard Deviations of the Study Variables in (A) Sample 1, (B) Sample 2

Variable	1	2	3	4	5	6	7	8	9	10	11	12	13	14	15	16	17
*(a)*																	
Female^a^																	
Schoolwork engagement 1	.01																
Schoolwork engagement 2	−.05	.82**															
School burnout 1	.04	−.69**	−.59**														
School burnout 2	.13**	−.48**	−.63**	.77**													
Curiosity 1	.01	.54**	.47**	−.36**	−.28**												
Curiosity 2	−.08	.49**	.53**	−.34**	−.28**	.47**											
Grit 1	−.09	.44**	.39**	−.37**	−.30**	.50**	.32**										
Grit 2	−.20**	.42**	.47**	−.38**	−.36**	.36**	.54**	.44**									
Academic buoyancy 1	−.22**	.28**	.26**	−.33**	−.32**	.27**	.16**	.40**	.27**								
Academic buoyancy 2	−.36**	.32**	.40**	−.42**	−.48**	.21**	.30**	.29**	.43**	.42**							
Social engagement 1	.17**	.37**	.30**	−.21**	−.14**	.52**	.31**	.44**	.27**	.24**	.09*						
Social engagement 2	.10	.35**	.35**	−.22**	−.18**	.33**	.46**	.24**	.38**	.12**	.23**	.41**					
Loneliness 1	.14**	−.20**	−.19**	.38**	.36**	−.12**	−.11*	−.19**	−.18**	−.26**	−.22**	−.10*	−.09				
Loneliness 2	.30**	−.24**	−.27**	.39**	.44**	−.15**	−.10**	−.22**	−.24**	−.27**	−.34**	−.09	−.09*	.49**			
Belongingness 1	−.09	.25**	.21**	−.29**	−.20**	.22**	.15**	.32**	.26**	.31**	.19**	.29**	.21**	−.49**	−.37**		
Belongingness 2	−.22**	.25**	.27**	−.28**	−.30**	.17**	.17**	.22**	.30**	.28**	.33**	.17**	.25**	−.37**	−.67**	.47**	
Mean		0.01	−0.41	0.00	0.15	2.73	2.72	3.40	3.42	4.57	4.61	2.75	2.78	1.72	1.99	4.57	3.94
*SD*		2.30	2.41	1.30	1.45	0.65	0.67	0.84	0.83	1.48	1.64	0.65	0.64	0.92	1.03	1.16	0.96

*(b)*																	
Female^a^																	
Schoolwork engagement 1	−.02																
Schoolwork engagement 2	−.06	.82**															
School burnout 1	.16**	−.64**	−.54**														
School burnout 2	.26**	−.45**	−.60**	.77**													
Curiosity 1	.02	.53**	.47**	−.32**	−.26**												
Curiosity 2	.00	.46**	.51**	−.28**	−.27**	.52**											
Grit 1	−.07	.49**	.42**	−.38**	−.29**	.54**	.36**										
Grit 2	−.10	.42**	.50**	−.34**	−.36**	.33**	.57**	.44**									
Academic buoyancy 1	−.38**	.32**	.28**	−.38**	−.38**	.23**	.08	.39**	.22**								
Academic buoyancy 2	−.43**	.28**	.33**	−.42**	−.49**	.07	.16**	.21**	.34**	.47**							
Social engagement 1	.19**	.37**	.34**	−.17**	−.13**	.53**	.37**	.45**	.22**	.16**	.04						
Social engagement 2	.22**	.31**	.29**	−.15**	−.07	.35**	.46**	.31**	.36**	.06	.07	.47**					
Loneliness 1	.28**	−.21**	−.22**	.35**	.32**	−.10*	−.09	−.21**	−.24**	−.34**	−.30**	−.12**	−.04				
Loneliness 2	.42**	−.15**	−.19**	.33**	.38**	−.05	−.11**	−.13**	−.22**	−.30**	−.42**	.00	−.09*	.55**			
Belongingness 1	−.15**	.23**	.21**	−.23**	−.18**	.19**	.17**	.29**	.23**	.28**	.23**	.26**	.16**	−.53**	−.35**		
Belongingness 2	−.30**	.18**	.24**	−.23**	−.26**	.09	.17**	.20**	.31**	.24**	.37**	.15**	.26**	−.44**	−.67**	.49**	
Mean		−0.09	−0.50	0.51	0.69	2.68	2.74	3.31	3.40	4.18	4.21	2.66	2.78	2.02	2.21	4.26	3.72
*SD*		2.11	2.18	1.24	1.25	0.67	0.67	0.83	0.81	1.58	1.63	0.67	0.62	1.04	1.04	1.26	0.96

SD, standard deviation.

^*^ *p* < .005, ^**^ *p* < .001.

^a^ Categorical.

#### School engagement

School engagement was measured using a short version of the schoolwork engagement inventory (Salmela‐Aro & Upadyaya, 2012) measuring energy, dedication, and absorption at school. The responses were rated on a 7‐point scale (1 = *never*; 7 = *daily*). The reliabilities in sample 1 were (Cronbach’s α_T1_ = .86, α_T2_ = .87; McDonald’s Ω_T1_ = .87, Ω_T2_ = .88) and in sample 2 (α_T1_ = .86, α_T2_ = .87; Ω_T1_ = .86, Ω_T2_ = .87).

#### School burnout

School Burnout was examined with the short version of School Burnout Inventory (Salmela‐Aro, Kiuru, Leskinen, & Nurmi, 2009) consisting of five items measuring school burnout: feelings of exhaustion, cynicism, and sense of inadequacy at school. The responses were rated on a 6‐point scale (1 = *strongly disagree;* 6 = *strongly agree*). The reliabilities were (α_T1_ = .84, α_T2_ = .88; Ω_T1_ = .84, Ω_T2_ = .88) for elementary school, and (α_T1_ = .86, α_T2_ = .85; Ω_T1_ = .86, Ω_T2_ = .85) for middle school.

#### Socio‐emotional skills

Socio‐emotional skills were examined on the basis of OECD (Kankaras & Suarez‐Alvarez, 2019) framework in terms of curiosity, grit, academic buoyancy, social engagement, loneliness, and social belongingness. *Curiosity* was measured using Epistemic Curiosity Scale (Litman, 2008) with five items (e.g., *“I find it fascinating to learn new information.”;* sample 1 α_T1_ = .84, α_T2_ = .88; Ω_T1_ = .84, Ω_T2_ = .88; sample 2 α_T1_ = .89, α_T2_ = .89; Ω_T1_ = .89, Ω_T2_ = .89) rated on a 4‐point scale (1 = *Almost never*; 4 = *Almost always*). *Grit* was measured using the short version of the grit scale (Duckworth et al., 2007) measuring perseverance of effort with three items (i.e., “*Setbacks don’t discourage me*.”; sample 1 α_T1_ = .73, α_T2_ = .75; Ω_T1_ = .75, Ω_T2_ = .76; sample 2 α_T1_ = .73, α_T2_ = .74; Ω_T1_ = .76, Ω_T2_ = .76). The responses were rated on a 5‐point scale (1 = *Not at all like me*, to 5 = *Very much like me*). *Academic buoyancy* (Martin & Marsh, 2008) was measured with three items (i.e., *“I don't let study stress get on top of me. “*sample 1 α_T1_ = .80, α_T2_ = .88; Ω_T1_ = .81, Ω_T2_ = .88; sample 2 α_T1_ = .87, α_T2_ = .90; Ω_T1_ = .87, Ω_T2_ = .90). The responses were rated on a 7‐point scale (1 = *strongly disagree*; 7 = *strongly agree*). *Social engagement* (Wang, Fredricks, Ye, Hofkens, & Linn, 2016) was measured with three items (i.e., *“I build on others' ideas.”*; sample 1 α_T1_ = .79, α_T2_ = .81; Ω_T1_ =.79, Ω_T2_ = .81; sample 2 α_T1_ = .84, α_T2_ = .82; Ω_T1_ = .84, Ω_T2_ = .82). The responses were rated on a 4‐point scale (1 = *Not at all* – 4 = *Extremely*). *Loneliness* was measured with a short version of UCLA Loneliness Scale (Russell, 1996) with three questions (i.e., *“How often do you feel that you lack companionship?”;* sample 1 α_T1_ = .91, α_T2_ = .93; Ω_T1_ = .91, Ω_T2_ = .93; sample 2 α_T1_ = .93, α_T2_ = .92; Ω_T1_ = .93, Ω_T2_ = .92). Students rated their answers with a 4‐point scale (1 = *Very rarely or never*; 4 = *Very often or always*). *Social belongingness* was measured in line with previous studies (Lappalainen & Hotulainen, 2012) using three items (i.e., *“I am doing really well with friends”*; sample 1 α_T1_ = .88, α_T2_ = .90; Ω_T1_ = .88, Ω_T2_ = .90; sample 2 α_T1_ = .89, α_T2_ = .90; Ω_T1_ = .89, Ω_T2_ = .90). The answers were rated with a 5‐point scale (1 = *Not at all*; 5 = *Completely*).

*Socioeconomic status* of the families was measured by asking students to rate their family’s financial situation with a scale from 1 = *Poor* to 5 = *Good*. *Gender* was coded 1 = female; 2 = male; 3 = other.

## ANALYSIS STRATEGY

As preliminary analyses, we filtered out as outliers participants who responded using only the highest or lowest extreme in all items in both school engagement and burnout. Missing data and attrition analyses were conducted. In total, there were 25% and 29% missing values in sample 1 and sample 2, respectively.

The pattern of missing values in the items was tested in both samples and resulted that values were missing completely at random in sample 1, χ^2^(8378) = 1254, *p* = 1, and in sample 2, χ^2^(6881) = 4332, *p* = 1. Missing values in the school engagement and burnout items were handled with Full information maximum likelihood in the analyses and the missing values for the auxiliary variables were handled with multiple imputation. Participant dropout after the first measurement was 23% in sample 1 and 17% in sample 2. In addition, there were 15% new participants at time 2 in sample 1 and 12% in sample 2. Based on binary logistic regression models the study variables did not predict dropout or drop in, and thus did not seem to compromise the findings. Measurement invariance of the school engagement and burnout measurement model was tested to ensure that the constructs carried the same meaning over groups and time, and the factor scores from the scalar invariance model were used as basis for the latent profile analyses (e.g., Morin, Meyer, Creusier, & Biétry, 2016). See supplementary file for details.

To answer the research questions, we adopted a latent profile analysis approach for the repeated measures of engagement and burnout. The models included engagement and burnout scores and the latent profiles were estimated to model the simultaneous change in both, that is, the profiles conceptually represented joint change trajectories of engagement and burnout between the two timepoints. The profiles were first estimated separately for each group, after which tests of profile similarity were conducted (Morin et al., 2016).

After closing on the final profile solution, we then examined how the groups would be characterized by simultaneous change and stability in the socio‐emotional skills by examining the within‐class latent change and rank‐order stability. Change accompanied with a high rank‐order stability would indicate that all participants in a certain class would show similar mean development, whereas a change accompanied with low rank‐order stability would indicate that they follow different patterns of change—that is, their relative standing at time 2 cannot be well predicted by their time 1 values. In the results, change refers to the latent change, whereas instability and stability are used to refer to low (or non‐significant) vs. medium to high rank‐order stability. In the analyses statistical significance was evaluated with the more conservative alpha of *p* < .005 (Benjamin et al., 2018) and Bonferroni correction was applied in the case of multiple testing.

## RESULTS

### Latent Profile Analyses

**Figure 1:**
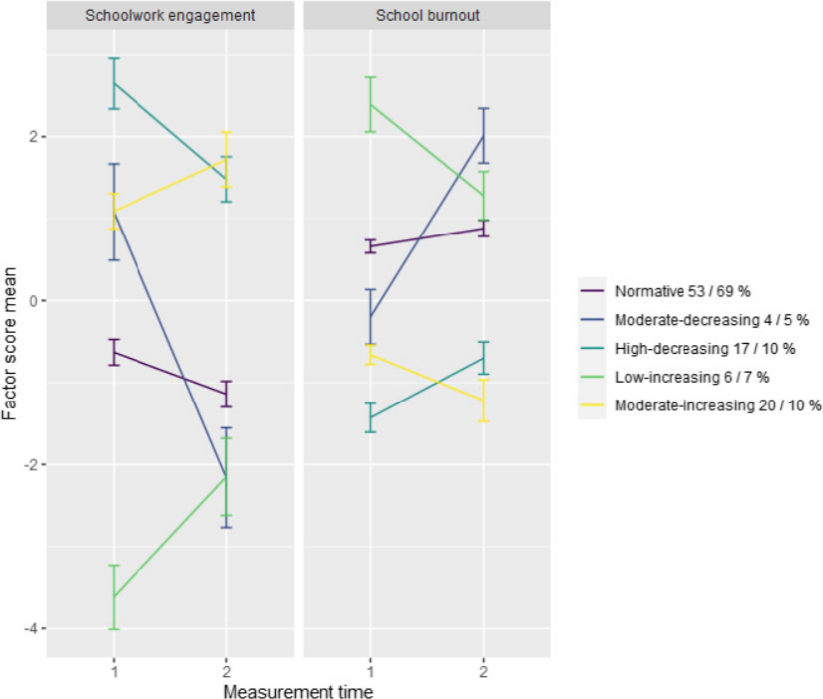
Engagement and burnout change pattern profiles.

Five profiles were considered to provide the best combination of model fit and substantive information in both samples. Following the tests of latent profile similarity, the model comparisons indicated that the structural similarity holds for the two samples, indicating that the means of the change patterns are similar enough in both samples (See Figure 1). The variances and profiles sizes differed. Further analyses were conducted on the structurally invariant two‐group five‐profile solution.

The profiles were labelled normative (53% sample 1; 69% sample 2), moderate‐decreasing (4%; 5%), high‐decreasing (17%; 10%), low‐increasing (6%;7%) and moderate‐increasing (20%; 10%) (the profile proportions do not add up precisely to 100 due to rounding). As can be seen from Table 2, all profiles showed statistically significant change. Compared to the normative profile, high‐decreasing or moderate‐increasing, there was slightly higher probability of a moderate‐decreasing participant to be female, whereas moderate‐increasing were least likely to be female. The gender distribution showed similarity across samples.

**Table 2:** Class Means and Within‐Class Mean Change in School Engagement and Burnout

		School Engagement						
		Time 1		Time 2				
	Profile	Mean	*SE*	Mean	*SE*	ΔMean	*SE*	*p*
1	Normative	−0.63	.08	−1.14	.08	−0.51	.03	<.001
2	Moderate‐decreasing	1.08	.30	−2.16	.31	−3.24	.23	<.001
3	High‐decreasing	2.65	.16	1.48	.14	−1.17	.09	<.001
4	Low−increasing	−3.62	.20	−2.15	.24	1.46	.18	<.001
5	Moderate‐increasing	1.09	.11	1.72	.17	0.63	.11	<.001

		School Burnout						
		Time 1		Time 2				
	Profile	Mean	*SE*	Mean	*SE*	ΔMean	*SE*	*p*
1	Normative	0.66	.04	0.88	.05	0.22	.02	<.001
2	Moderate‐decreasing	−0.20	.17	2.01	.17	2.21	.13	<.001
3	High‐decreasing	−1.43	.09	−0.70	.10	0.72	.04	<.001
4	Low‐increasing	2.39	.17	1.28	.15	−1.11	.13	<.001
5	Moderate‐increasing	−0.66	.06	−1.22	.13	−0.56	.10	<.001

Note

Alpha level.005/5 = .001.

*SE,* standard error.

### Within‐Class Change and Stability in Socio‐Emotional Skills

**Figure 2:**
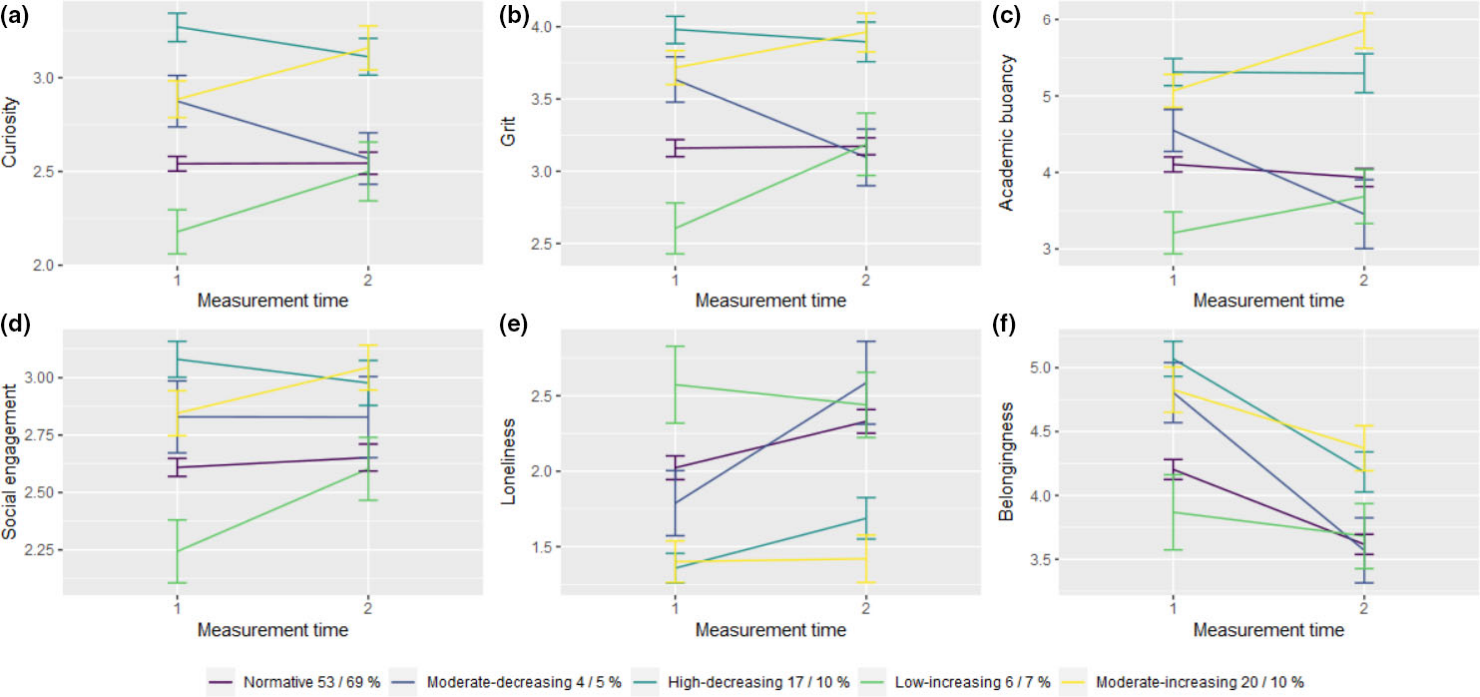
Latent differences across socio‐emotional competencies by profile (BCH).

[Figure 2 auxiliary variables by profile].

As can be inferred from Figure 2 as well as Table 3 the largest normative profile showed little to no changes in most socio‐emotional outcomes, although they did show a moderately small increase in loneliness and a decrease in belongingness alongside the other groups.

**Table 3:** Class‐Specific T1 Means, Latent Change and Rank‐Orders Stability in Auxiliary Variables

					Latent Change				Rank‐Order Stability		
	Profile	Variable	Mean T1	*SE*	ΔMean	*SE*	*p*	*SMD*	*r*	*SE*	*p*
1	Normative	Curiosity	2.54	.02	0.00	.03	.911	0.01	.42	.03	<.001
2	Moderate‐decreasing		2.88	.07	2.31	.07	<.001	−0.47	.42	.08	<.001
3	High‐decreasing		3.27	.04	−0.16	.05	.002	−0.25	.42	.07	<.001
4	Low‐increasing		2.18	.06	0.32	.08	<.001	0.50	.38	.08	<.001
5	Moderate‐increasing		2.89	.05	0.27	.06	<.001	0.42	.42	.08	<.001
1	Normative	Grit	3.16	.03	0.01	.03	.709	0.02	.36	.03	<.001
2	Moderate‐decreasing		3.64	.08	−0.54	.10	<.001	−0.63	.27	.08	.001
3	High‐decreasing		3.98	.05	−0.09	.07	.184	−0.10	.34	.07	<.001
4	Low‐increasing		2.61	.09	0.58	.11	<.001	0.68	.26	.08	.001
5	Moderate‐increasing		3.72	.06	0.25	.07	<.001	0.29	.39	.09	<.001
1	Normative	Academic buoyancy	4.11	.05	−0.17	.06	.006	−0.11	.39	.04	<.001
2	Moderate‐decreasing		4.55	.14	−1.09	.23	<.001	−0.68	.24	.09	.005
3	High‐decreasing		5.31	.09	−0.02	.13	.909	−0.01	.37	.07	<.001
4	Low‐increasing		3.21	.14	0.47	.18	.009	0.30	.35	.09	<.001
5	Moderate‐increasing		5.07	.11	0.79	.12	<.001	0.50	.35 (−.77)	.11 (.47)	.001 (.099)
1	Normative	Social engagement	2.61	.02	0.04	.03	.106	0.06	.41	.03	<.001
2	Moderate‐decreasing		2.83	.08	0.00	.09	.991	0.00	.29	.10	.005
3	High‐decreasing		3.08	.04	−0.10	.05	.056	−0.15	.31	.07	<.001
4	Low‐increasing		2.24	.07	0.36	.07	<.001	0.53	.43	.07	<.001
5	Moderate‐increasing		2.85	.05	0.20	.05	<.001	0.29	.45	.06	<.001
1	Normative	Loneliness	2.02	.04	0.31	.04	<.001	0.32	.48	.03	<.001
2	Moderate‐decreasing		1.79	.11	0.80	.14	<.001	0.82	.27	.10	.008
3	High‐decreasing		1.36	.05	0.33	.07	<.001	0.34	.57	.08	<.001
4	Low‐increasing		2.57	.13	−0.13	.11	.239	−0.14	.49	.06	<.001
5	Moderate‐increasing		1.40	.07	0.02	.08	.808	0.02	.51	.11	<.001
1	Normative	Belongingness	4.20	.04	−0.59	.04	<.001	−0.53	.43	.03	<.001
2	Moderate‐decreasing		4.81	.12	−1.24	.13	<.001	−1.12	.43	.09	<.001
3	High‐decreasing		5.07	.07	−0.89	.08	<.001	−0.80	.49	.07	<.001
4	Low‐increasing		3.87	.15	−0.19	.13	.150	−0.17	.53	.06	<.001
5	Moderate‐increasing		4.83	.09	−0.46	.09	<.001	−0.41	.52	.08	<.001

Note

Alpha level .005/5 = .001.

*SE*, standard error; SMD, standardized mean difference, *r*, correlation coefficient.

The profiles with an increasing trajectory showed a moderately stable and moderately large increases in intrapersonal socio‐emotional competencies curiosity, grit and academic buoyancy, as well as a moderately stable increase in social engagement. Most notably they showed no change in loneliness and either no change or less negative change in belongingness. The low‐increasing profile seemed especially characterized by a moderate to high increase in grit and the moderate‐increasing profile seemed to show a larger increase in academic buoyancy.

The decreasing profiles, in turn, showed the opposite pattern. The moderate‐decreasing profile, which showed the most detrimental trajectory in terms of engagement and burnout, showed also moderate to large, but less stable decreases in intrapersonal socio‐emotional competencies, no change in social engagement and large increase in loneliness and a large decrease in belongingness. The high‐decreasing profile showed a moderate increase in loneliness and a large decrease in belongingness with no changes in the other outcomes.

## DISCUSSION

This study took a person‐oriented approach in students’ academic well‐being and socio‐emotional skills during COVID‐19, and examined the latent profiles of study‐related engagement and burnout (e.g., exhaustion, cynicism, and feelings of inadequacy), as well as the simultaneous role of key socio‐emotional skills. Importantly, the present study had data available concerning the students’ baseline burnout, engagement, and socio‐emotional skills, which made it possible to compare students’ academic well‐being before and during the COVID‐19 pandemic.

Five academic well‐being change profiles were identified both among early and middle adolescents, of which the majority (53% and 69%) showed a normative slight decline in academic well‐being as would be developmentally expected (Salmela‐Aro et al., 2018). Interestingly, study engagement seemed to be impacted more. However, two of the profiles showed a steeper decrease (4–17%) and another two showed an increase (6–20%) in academic well‐being supporting H1. Thus, 74% of elementary students showed decrease in academic well‐being, whereas 26% of them experienced increases in academic well‐being during COVID‐19, and 84% of the middle school students showed decreases in academic well‐being, whereas among 16% of them academic well‐being increased during COVID‐19 supporting H2. Surprisingly, a large amount of students (17% and 10%) who reported decreases in their academic well‐being initially reported higher engagement than any other profiles, which, however, decreased during the COVID‐19 pandemic. These results need to be taken seriously as school burnout increases the risk of dropping out fourfold (Bask & Salmela‐Aro, 2013).

The findings also indicated that polarization occurred in students’ academic well‐being during COVID‐19. At time 2, the different well‐being change profiles became more similar, and students initially starting from different levels reported similar levels of engagement and burnout. As a consequence, most students reported either well‐being or ill‐being at Time 2 (Figure 1). It is, however, and empirical question worthy of further studies to examine whether the patterns stabilized in the polarized situation or continued increasing or decreasing further during the continuing pandemic. For many students, constant stress is the new normal during the COVID‐19 pandemic (Hoyt et al., 2021), and various fears and worries about the pandemic, and social, psychological, and financial turmoil that the COVID‐19 triggered may further amplify these negative changes in students’ well‐being if the situation persists. Thus, the negative changes in academic well‐being might have already continued developing since the last data collection of the present study, showing as further decreases in students’ academic well‐being. Thus, it would be of uttermost importance for researchers, educators, and policymakers to react fast and develop ways to promote student well‐being and decrease their burnout symptoms.

Overall, regarding the interplay between socio‐emotional competencies it seems that they develop somewhat in conjunction supporting H3, that is, change and stability in engagement and burnout were accompanied with change or stability in key socio‐emotional skills. More precisely, increasing study‐related well‐being were reflected also in experiences of curiosity, grit or academic buoyancy, whereas decreasing well‐being was more strongly linked with social factors such as social engagement, loneliness, and belongingness (Schoon, 2021). As can be inferred from Figure 2 as well as Appendix C the largest normative group showed little to no changes in most socio‐emotional outcomes, although they did show a moderately small increase in loneliness and a decrease in belongingness alongside the other groups.

However, in the moderate‐decreasing and high‐decreasing profiles loneliness increased and belongingness decreased to a large extent, which is a concerning finding. Other studies have reported that especially students are in altered risk for loneliness during COVID‐19 (Bu, Steptoe, & Fancourt, 2020). School is often the main environment where students’ social relationships exist, however, during the school closures and social distancing measures social relationships reduced dramatically, showing as small increases in loneliness even among those students who otherwise were doing well and/or were highly engaged in school.

On the positive side, among approximately every fourth of the students’ engagement increased during COVID‐19. What seemed to distinguish them was that they experienced moderate stability or increases in their socio‐emotional skills during these years. Instead, they were able to employ curiosity and grit. These results show the importance of the socio‐emotional skills in pandemic, and that social factors seem to be key components for engagement and burnout. Socio‐emotional competences are relevant in protecting students exposed crisis situations, such as COVID‐19 pandemic, to thrive (Schoon, 2021).

## LIMITATIONS

This study has some limitations. First, the study was conducted among Finnish adolescents which should be taken into account in generalizing the results. More similar studies would be needed examining students’ school‐related well‐being, demands and resources, such as socio‐emotional skills at different cultural context and grade levels. However, although this paper focuses on Finland, the results are largely generalizable to other countries. In PISA surveys, 15‐year‐old students in Finland have outperformed their peers in other countries. However, the PISA results also show that students in Finland are not happy at school. For young people, positive school adaptation is a precursor of their future adaptation, and hence school burnout may have negative, possibly cascading, future consequences. In addition, interventions should not aim only reducing school burnout but also increasing school engagement; a problem faced in many educational systems.

## CONCLUSIONS

The present findings showed the importance of socio‐emotional skills in promoting students’ academic well‐being. To better support students who are at risk for burnout during pandemic, school psychologist could help students to create coping strategies to deal with stressors (Styck et al., 2020), help students interpret information accurately concerning the pandemic, and to manage their emotions and behaviors in a beneficial way in order to reduce their stress and burnout symptoms (Haig‐Ferguson, Cooper, Cartwright, Loades, & Daniels, 2020).

Schools and educational institutions should also consider who are the most vulnerable populations to suffer anxiety, stress, and burnout (Hoyt et al., 2021). For example, students who were already in a vulnerable situation prior to the pandemic often had reduced access to support and resources (e.g., special education tutoring, school psychologist). Also in Finland immigrants suffered more of COVID‐19 cases compared to the natives, and students coming from immigrant families might have experienced increased stress and fear concerning the spread of the virus. The results also indicated that loneliness increased during the pandemic in both student populations, which is concerning. Many interventions to stop COVID‐19 from spreading have included some form of social isolation, which has resulted as increased loneliness among students. Loneliness, in turn, may manifest as sleep problems and further decreases in well‐being (Groarke et al., 2020). It would be of great importance to find ways to cope with the pandemic without creating new health concerns (see also Hoyt et al., 2021). Interventions focusing on better emotion regulation and social support may help in reducing loneliness (Groarke et al., 2020) .

## Supporting information

File S1. Adolescents' longitudinal school engagement and burnout before and during COVID‐19 – The role of socio‐emotional skills
