# Supplementary material for: Adolescents' Longitudinal School Engagement and Burnout Before and During COVID‐19—The Role of Socio‐Emotional Skills
Source: J Res Adolesc. 2021 Aug 26;31(3):796–807. doi: 10.1111/jora.12654 (PMC8646577; doi:10.1111/jora.12654)
Supplement: Supplementary file 1 — File S1. Adolescents' longitudinal school engagement and burnout before and during COVID‐19 – The role of socio‐emotional skills [file JORA-31--s001.docx]

ADDITIONAL MATERIAL FOR

**Adolescents' longitudinal school engagement and burnout before and during COVID-19 –**

**The role of socio-emotional skills**

Contents

[**Data and code** 1](#_Toc73619004)

[**Missing values and attrition** 1](#_Toc73619005)

[Sample 1 1](#_Toc73619006)

[Sample 2 4](#_Toc73619007)

[**Preliminary measurement models** 7](#_Toc73619008)

[**Tests of Latent Profile Similarity** 8](#_Toc73619009)

[**References** 11](#_Toc73619010)

## **Software**

All analyses were conducted with Mplus 8.5 (Muthén & Muthén, 1998-2020) and R 4.0.3 with Rstudio 1.3.1073 ([www.rstudio.com](http://www.rstudio.com/)) and packages MplusAutomation (Hallquist & Wiley, 2018) as well as set of packages and tools from the Tidyverse -collection (Wickham et al., 2019) and userfriendlyscience (Peters, 2018).

## **Data and code**

Data and materials to reproduce the results, as well as additional results can be downloaded from <https://osf.io/s6rkq/?view_only=11672f256b754c4c9977a0b369e5c0a5>.

## **Missing values and attrition**

**Preliminary analyses**

After filtering out outliers, there were 1381 participants in sample 1 and 1374 participants in sample 2. Participant dropout after the first measurement was 23% in sample 1 and 17% in sample 2. In addition, there were 15% new participants at time 2 in sample 1 and 12% in sample 2. Based on binary logistic regression models the study variables did not predict dropout or drop in.

In the measurement models the missing data were handled with Full information maximum likelihood (FIML) and when comparing profiles across auxiliary variables the missing data on the auxiliary variables were multiply imputed. Both of these approaches are recommended instead or data-deletion and are generally regarded as equally effective (e.g. Dong & Peng, 2013). The chapters below describe the missing data and attrition analyses for each sample.

### Sample 1

There were altogether 25% missing in the items used in the analyses, that were missing completely at random (χ^2^(8378)=1254, *p* = 1). Most of the missing values were a product of students missing the whole questionnaire in either time point or given that the questionnaire were collected in two parts – missing the other. In timepoint 1 there were 12.2% data missing from the items and in timepoint 2 there were only 5.9% missing from the items. Figure A1 depicts the pattern of missing values for all items, Figure A2 the school engagement and burnout items used in examining the measurement model.


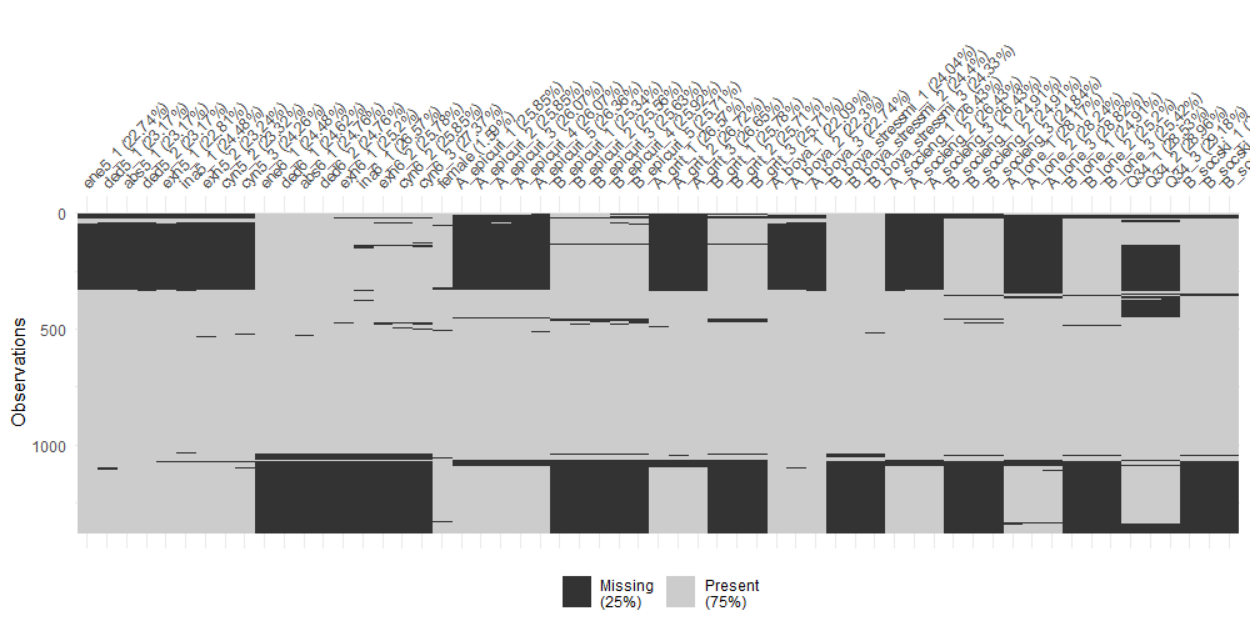
**Figure A1**. Missing values in Sample 1 school engagement and burnout items


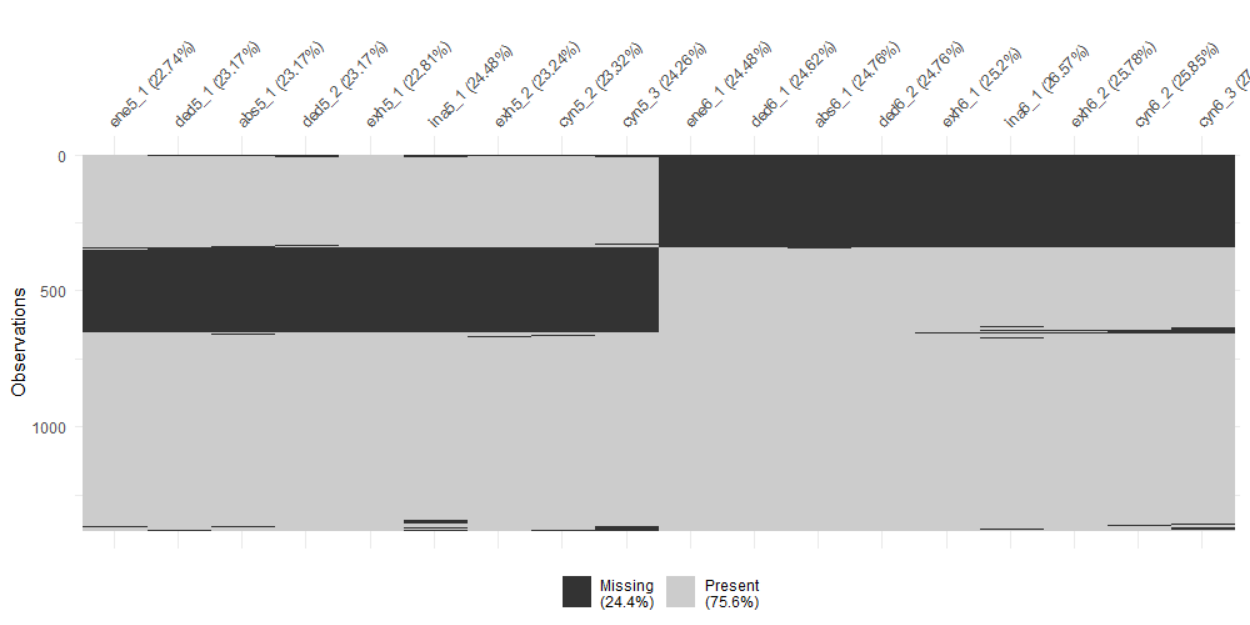
**Figure A2.** Missing values in Sample 1 school engagement and burnout items

Attrition-wise there were 319 participants dropping out after the first measurement occasion, a binary logistic regression model indicated that the dropout was not well predicted by either school engagement, burnout or the socio-emotional competencies (no significant predictors at p<.005; Nagelkerke R^2^ = 0.03).


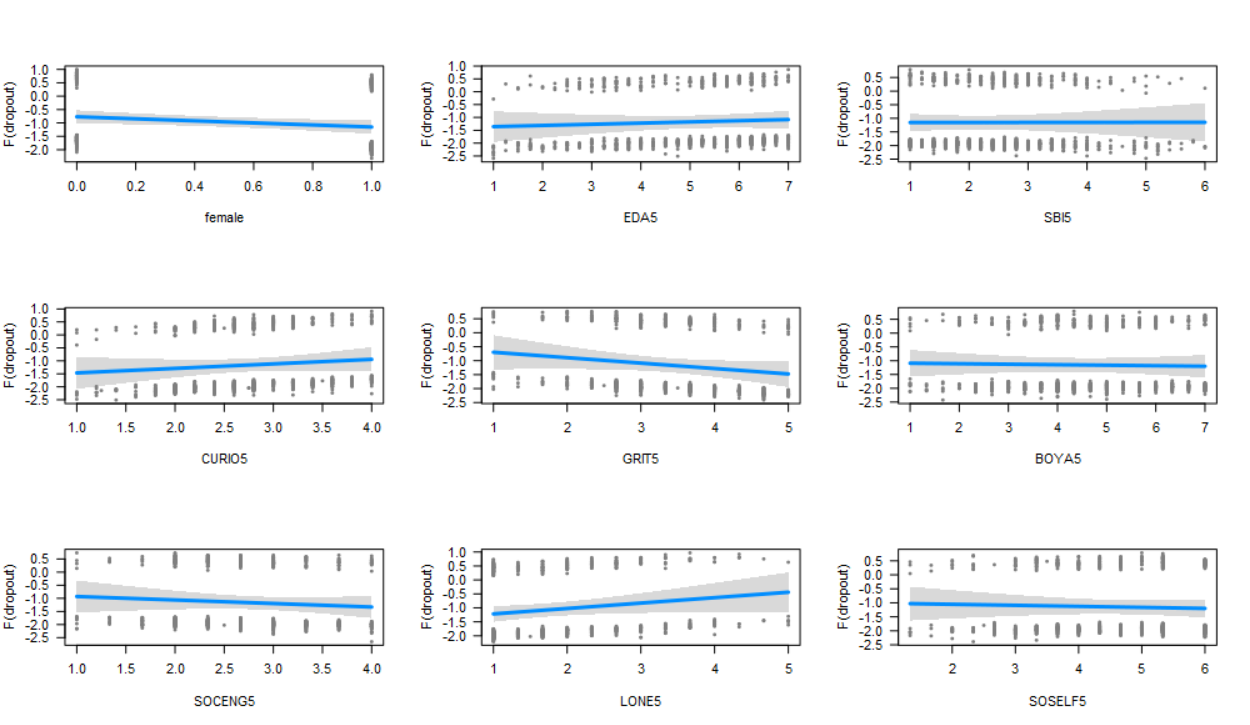
**Figure A3.** The probability to dropout predicted by time 1 variables


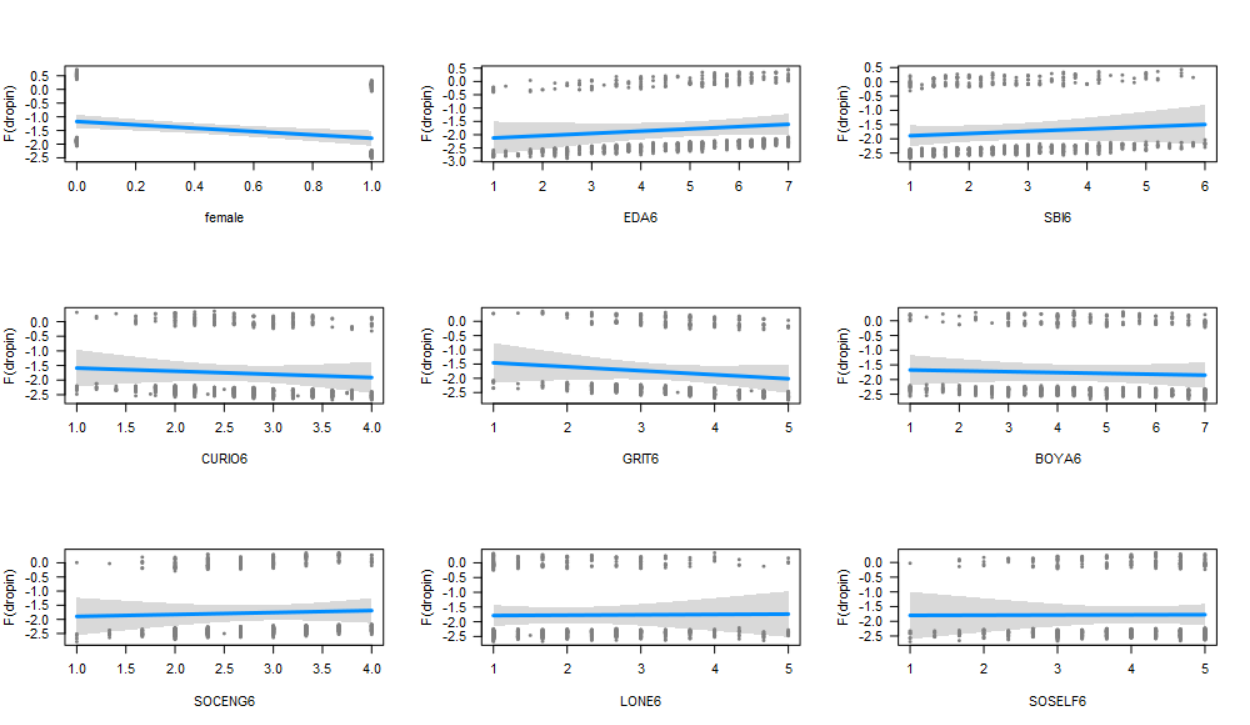
**Figure A4.** The probability to dropin predicted by time 2 variables

In addition there were 209 new participants at time 2, a binary logistic regression model indicated that the new participants were not different (Nagelkerke R^2^ = 0.02) from the main longitudinal sample with the exception that the new participants were slightly more likely to be male students. Thus, attrition and drop-in did not seem to compromise the findings.

### Sample 2

There were altogether 29% missing in the items used in the analyses, that were missing completely at random (χ^2^(6881)=4332, *p* = 1). Most of the missing values were a product of students missing the whole questionnaire in either time point or given that the questionnaire were collected in two parts – missing the other. In timepoint 1 there were 15.8% data missing from the items and in timepoint 2 there were only 6.5% missing from the items. Figure B1 depicts the pattern of missing values for all the items, Figure B2 the school engagement and burnout items used in examining the measurement model.


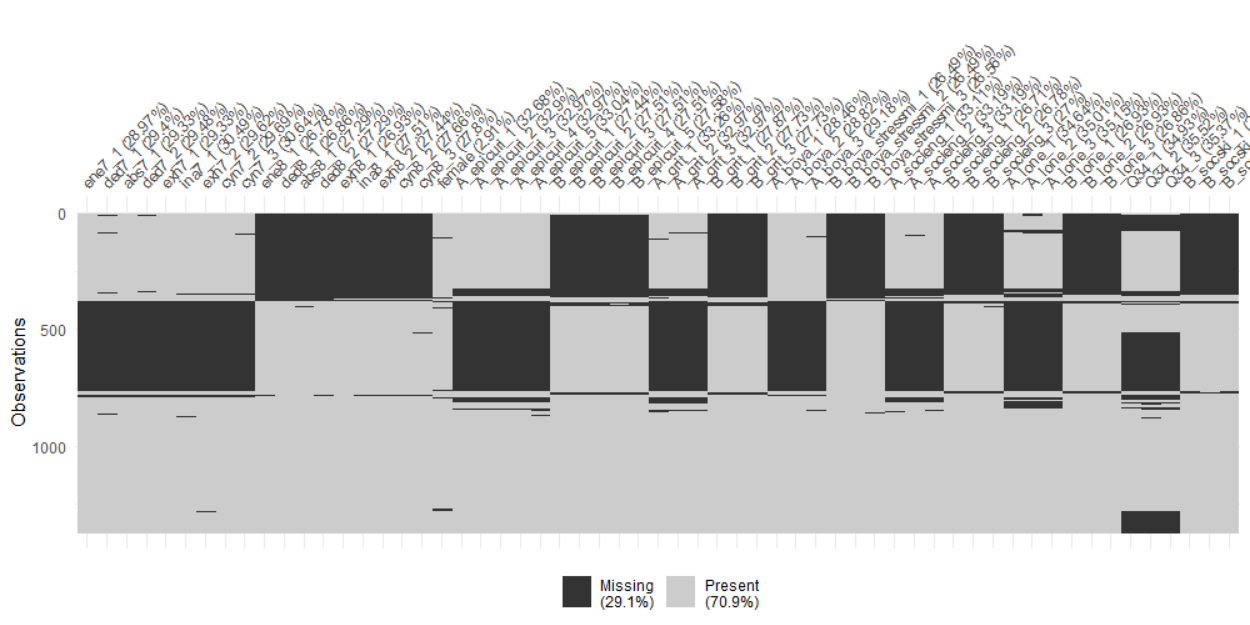
**Figure B1.** Missing values in Sample 2 all items


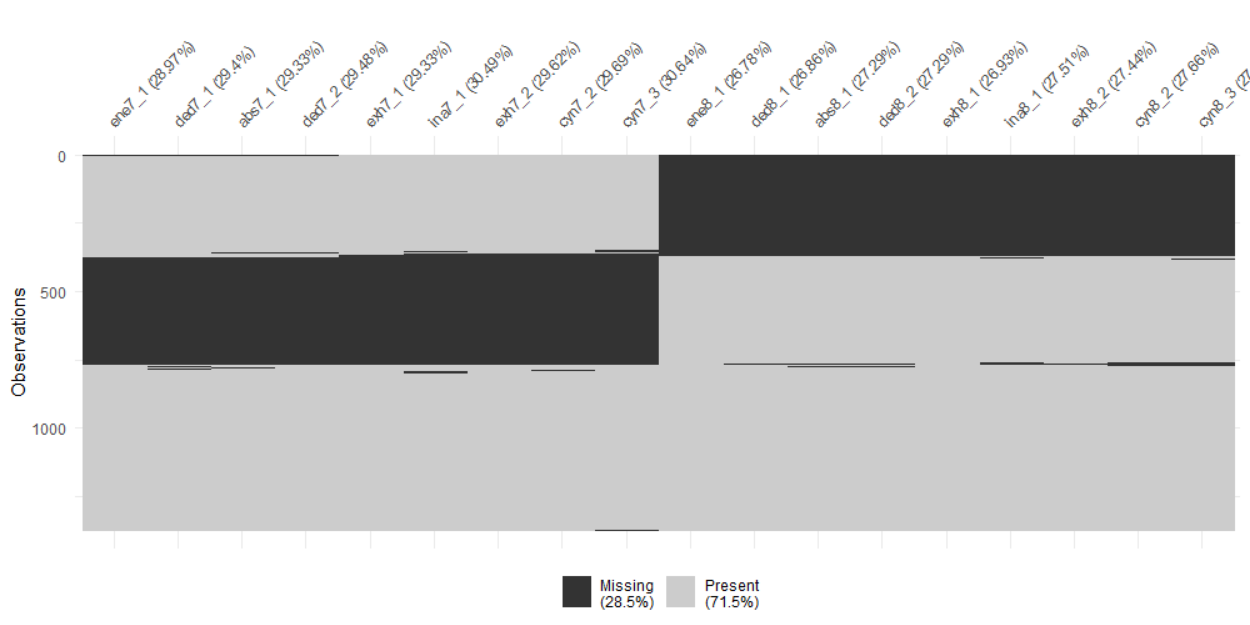
**Figure B2.** Missing values in Sample 2 school engagement and burnout items

Attrition-wise there were 233 participants dropping out after the first measurement occasion, a binary logistic regression model indicated that the dropout was not well predicted by either school engagement, burnout or the socio-emotional competencies as seen from Figure B3 (no significant predictors at p<.005; Nagelkerke R^2^ = 0.04).


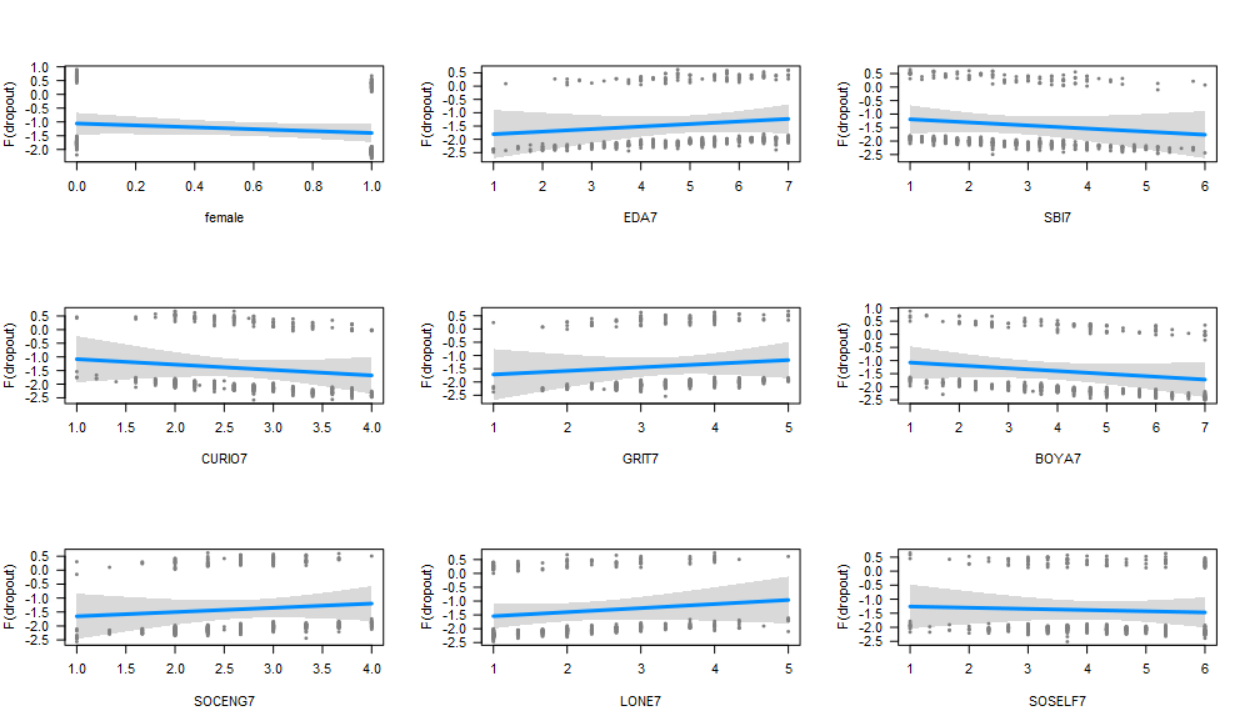
**Figure B3.** The probability to dropout predicted by time 1 variables


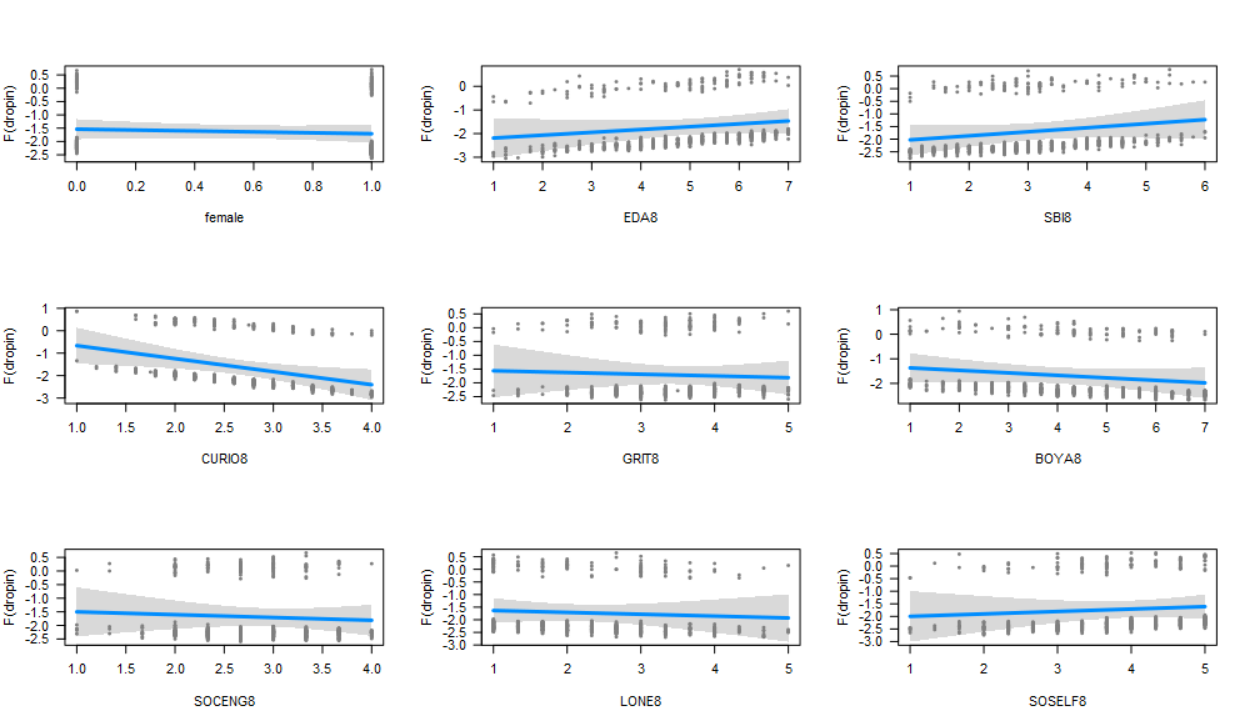
**Figure B4.** The probability to dropin predicted by time 2 variables

In addition there were 163 new participants at time 2, a binary logistic regression model indicated that the new participants were not different from the main longitudinal sample as seen from Figure B4 (no significant predictors; Nagelkerke R^2^ = 0.05). Thus, attrition and drop-in did not seem to compromise the findings.

## **Preliminary measurement models**

The measurement invariance tests were specified as longitudinal CFA-models. All models were specified with the Maximum likelihood estimator with robust standard errors and the nested structure of the data were taken into account with TYPE=COMPLEX in Mplus. Measurement invariance were tested for school engagement and burnout following the standard procedures of measurement invariance (see Putnick & Bornstein, 2016) testing for ordinal variables (see also Millsap & Tein, 2004, Muthén & Muthén, 1998-2020). That is, we tested for invariance of factor structure, factor loadings and factor thresholds over time and across samples using a multiple-group longitudinal confirmatory factor analysis model. The models were estimated with maximum likelihood with numerical integration and FIML were used with missing data.

**Table 1.** Multigroup longitudinal measurement invariance

| **Filename** | **Parameters** | **LogLikelihood** | **LL_CorrectionFactor_** | **BIC** | **aBIC** | **-2ΔLL** | **df** | ***p*** |
| --- | --- | --- | --- | --- | --- | --- | --- | --- |
| Configural | 231 | -56474.66 | 1.14 | 114779.10 | 114045.20 |  |  |  |
| Metric | 210 | -56522.82 | 1.14 | 114709.10 | 114041.80 | 80.33 | 21 | <.001 |
| Strong | 81 | -56687.39 | 1.36 | 114016.40 | 113759.00 | 327.99 | 129 | <.001 |

Invariance were tested with likelihood ratio chi-square difference tests (<https://www.statmodel.com/chidiff.shtml>) as well as by comparing BIC values, a lower BIC indicating a better balance of fit and parsimony. The model with invariant thresholds showed the lowest BIC across all comparisons. Based on the invariance tests strong (scalar) invariance was declared both across groups as well as longitudinally and factor scores were saved from the final multigroup model to be used in latent profile analyses.

**Table 2A.** Longitudinal measurement invariance for Sample 1

| **Filename** | **Parameters** | **LogLikelihood** | **LL_CorrectionFactor_** | **BIC** | **aBIC** | **-2ΔLL** | **df** | ***p*** |
| --- | --- | --- | --- | --- | --- | --- | --- | --- |
| Configural | 122 | -27511.07 | 1.09 | 55904.27 | 55516.72 |  |  |  |
| Metric | 115 | -27517.79 | 1.08 | 55867.10 | 55501.79 | 11.14 | 7 | 0.133 |
| Strong | 72 | -27575.94 | 1.13 | 55672.48 | 55443.77 | 116.10 | 43 | <.001 |

**Table 2B.** Longitudinal measurement invariance for Sample 2

| **Filename** | **Parameters** | **LogLikelihood** | **LL_CorrectionFactor_** | **BIC** | **aBIC** | **-2ΔLL** | **df** | ***p*** |
| --- | --- | --- | --- | --- | --- | --- | --- | --- |
| Configural | 122 | -27022.36 | 1.11 | 54926.24 | 54538.69 |  |  |  |
| Metric | 115 | -27032.38 | 1.11 | 54895.70 | 54530.39 | 16.13 | 7.00 | 0.024 |
| Strong | 72 | -27060.65 | 1.19 | 54641.54 | 54412.82 | 58.55 | 43.00 | 0.057 |

**Table 3.** Measurement model factor loadings and r^2^

|  | λ_EDA_ | λ_SBI_ | r^2^_5th_ | r^2^_6th_ | r^2^_7th_ | r^2^_8th_ |
| --- | --- | --- | --- | --- | --- | --- |
| ENE1 | 1.00 | 0 | 0.67 | 0.68 | 0.66 | 0.68 |
| DED1 | 0.90 | 0 | 0.62 | 0.64 | 0.62 | 0.63 |
| ABS1 | 0.69 | 0 | 0.49 | 0.51 | 0.49 | 0.50 |
| DED2 | 1.10 | 0 | 0.71 | 0.72 | 0.71 | 0.72 |
| EXH1 | 0 | 1.00 | 0.40 | 0.45 | 0.41 | 0.42 |
| INA1 | 0 | 1.36 | 0.55 | 0.61 | 0.57 | 0.57 |
| EXH2 | 0 | 1.28 | 0.52 | 0.58 | 0.54 | 0.54 |
| CYN2 | 0 | 1.92 | 0.71 | 0.75 | 0.72 | 0.73 |
| CYN3 | 0 | 1.22 | 0.50 | 0.55 | 0.51 | 0.52 |

## **Reliability analyses**

We estimated the internal consistencies and composite reliability of the measures utilized in the present study with both Cronbach’s Alpha and McDonald’s Omega. McDonald's Omega internal consistency reliability coefficients are based on the parameter estimates of the factor model and thus provides a more accurate estimate of multi-item measurement scale validity than Cronbach's Alpha (Hayes & Coutts, 2020) which is based on tau-equivalence, resulting in assumptions of unidimensionality and equal variances of and covariances between the items (Peters, 2018), which is often an untenable assumption. Thus, Omega is often considered to be a better estimate of composite reliability. For the sake of tradition and measurement accuracy both reliability estimates are reported in the paper.

## **Tests of Latent Profile Similarity**

Utilizing the factor scores saved from the latent measurement model the latent profile analyses (LPA) were first estimated separately for each sample to narrow down the number of profiles and as the first step of establishing *configural* similarity of profiles (for a discussion on tests of latent profile similarity see Morin et al., 2016). The repeated measures LPAs were specified so that the variables from both assessment occasions were entered into the same model and the conditional independence assumption was relaxed by allowing within-class residual covariance between the same variable across time.

Class enumeration was informed by first increasing the number of classes and examining change in information criteria including the Bayesian information criteria (BIC) and consistent Akaike’s information criteria (CAIC), the latter providing a stronger penalty for model complexity. These were selected given their stable performance across conditions (see e.g. Nylund et al., 2007; Tein et al., 2013). The number of classes were to be increased until the lowest BIC and CAIC would be reached, or a noticeable elbow (Masyn, 2013) after which the gains in model fit gained by additional classes could be considered negligible. After narrowing down the set of considered models with the joint information provided by the information criteria the competing neighboring models were then compared with Vuong-Lo-Mendell-Rubin (VLMR) and bootstrapped (BLRT) likelihood ratio tests (Asparouhov & Muthén, 2007) as well as the substantive quality and usefulness of the profile solution (Morin & Marsh, 2015). For a more thorough discussion on the LPA procedure see Ferguson et al. (2020).

Table 4 shows the information criteria for the compared model solutions. In both samples BIC, CAIC and BLRT failed to unambiguously support any solution, but BIC and CAIC showed a noticeable elbow (Figure 5) at six profiles in both samples and VLMR did not support six profiles over five. Solutions with four, five and six profiles were examined more closely. The models were statistically proper, yet the six profile solution in both samples estimated an additional very small (<5%) profile likely to be spurious and thus yielding little substantive value. The five-profile solutions were retained as first evidence and the baseline for configural similarity model.


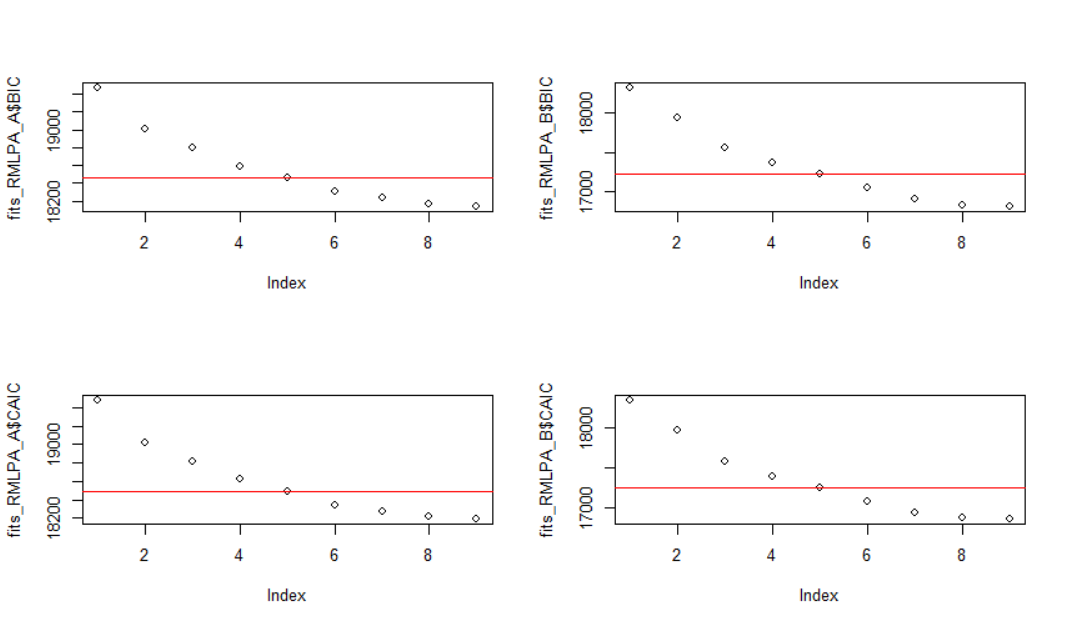
**Figure 5.** BIC and CAIC elbow plots

**Table 4.** Fit indices of separate latent profile analyses

|  |  | **Elementary sample** | | | | | **Secondary sample** | | | | |
| --- | --- | --- | --- | --- | --- | --- | --- | --- | --- | --- | --- |
| **Profiles** | **Parameters** | **LL** | **BIC** | **CAIC** | ***p*_VLMR_** | ***p*_BLRT_** | **LL** | **BIC** | **CAIC** | ***p*_VLMR_** | ***p*_BLRT_** |
| 1 | 10 | -9700.76 | 19473.82 | 19483.82 |  |  | -9128.8 | 18329.86 | 18339.86 |  |  |
| 2 | 15 | -9449.08 | 19006.62 | 19021.62 |  |  | -8917.57 | 17943.52 | 17958.52 |  |  |
| 3 | 20 | -9323.25 | 18791.10 | 18811.10 |  |  | -8707.79 | 17560.09 | 17580.09 |  |  |
| 4 | 25 | -9206.99 | 18594.75 | 18619.75 |  |  | -8595.34 | 17371.31 | 17396.31 |  |  |
| 5 | 30 | -9121.27 | 18459.46 | 18489.45 | 0.013 | <0.001 | -8502.93 | 17222.62 | 17252.62 | 0.022 | <0.001 |
| 6 | 35 | -9025.59 | 18304.26 | 18339.26 | 0.089 | <0.001 | -8400.46 | 17053.81 | 17088.81 | 0.066 | <0.001 |
| 7 | 40 | -8971.41 | 18232.04 | 18272.04 |  |  | -8306.87 | 16902.76 | 16942.76 |  |  |
| 8 | 45 | -8922.18 | 18169.74 | 18214.74 |  |  | -8253.12 | 16831.4 | 16876.39 |  |  |
| 9 | 50 | -8886.62 | 18134.76 | 18184.76 |  |  | -8223.67 | 16808.61 | 16858.61 |  |  |

The similarity of the class-specific factor score means were then compared in order to test for *structural* similarity. Specifically, this was done by comparing models in which the means were held equal and freely estimated. In the comparison we relied on BIC and CAIC. As can be seen from Table 5 the lowest BIC and CAIC were achieved with a model assuming structural similarity, indicating that the latent profiles shared similar mean structure but differing variances. We then used the MODEL CONSTRAINT in Mplus to examine whether the mean differences between time 1 and time 2 were statistically significant within-class with a multivariate delta method (Raykov & Marcoulides, 2004).

**Table 5.** Fit indices of separate latent profile analyses

| Model | Parameters | LL | BIC | CAIC |
| --- | --- | --- | --- | --- |
| Configural similarity | 59 | -19535.26 | 39537.86 | 39596.86 |
| Structural similarity | 39 | -19593.72 | 39496.36 | 39535.36 |
| Dispersion similarity | 35 | -19624.08 | 39525.39 | 39560.40 |

To compare the latent change profiles over simultaneous change in the socio emotional components we then utilized the manual BCH approach to adjust for the non-perfect classification accuracy (Asparouhov & Muthén, 2020) and held the structural similarity constraints in place. The auxiliary variables were multiply imputed over 100 datasets. In the third step of the three-step manual BCH approach we further tested for latent profile similarity over the latent changes in auxiliary variables. This approach is conceptually similar to what is presented in Morin et al (2016) as a test for *explanatory* similarity with the extension of using the BCH-weights to ensure that the profile solution would stay unaffected while simultaneously taking into account classification uncertainty.

However, given that our research question differed, we actually tested for whether the latent profiles were able to explain similar latent change and rank-order stability in the auxiliary variables over the two samples as well as whether this would differ across samples. This was tested by specifying a latent difference score model (see e.g. McArdle & Hamagami, 2001) with the means allowed to vary across profiles and groups and comparing it to a model in which the means were allowed to vary only across profiles. The decision of similarity was based on the pooled BIC and CAIC.

The results indicated that across all variables the model assuming that the latent change scores do not differ across samples fit the data better, and regarding the rank-order stabilities the results were the same with the exception of Academic buoyancy in which only partial similarity was supported.

**Table 6.** Pooled BIC values for the explanatory similarity tests

|  | **fp** | **Curiosity** | | **Grit** | | **Academic buoancy*** | |
| --- | --- | --- | --- | --- | --- | --- | --- |
|  |  |  |  |  |  |  |  |
|  |  | **BIC** | **CAIC** | **BIC** | **CAIC** | **BIC** | **CAIC** |
| Freely estimated latent change | 32 | 19742.45 | 19774.45 | 22337.35 | 22369.35 | 29439.83 | 29471.83 |
| Explanatory similarity | 22 | 19743.70 | 19765.70 | 22325.27 | 22347.27 | 29404.98 | 29426.98 |
|  |  |  |  |  |  |  |  |
| Freely estimated rank order stability | 41 | 19775.71 | 19816.71 | 22388.13 | 22429.13 | 29428.59 | 29469.59 |
| Explanatory similarity | 36 | 19771.44 | 19807.44 | 22361.12 | 22397.12 | 29405.31 | 29442.31 |
|  |  | **Social engagement** | | **Loneliness** | | **Belongingness** | |
|  |  |  |  |  |  |  |  |
|  |  | **BIC** | **CAIC** | **BIC** | **CAIC** | **BIC** | **CAIC** |
| Freely estimated latent change | 32 | 20348.19 | 20380.19 | 24644.94 | 24676.94 | 25672.25 | 25704.25 |
| Explanatory similarity | 22 | 20162.23 | 20184.23 | 24617.92 | 24639.92 | 25629.36 | 25651.36 |
|  |  |  |  |  |  |  |  |
| Freely estimated rank order stability | 41 | 20241.61 | 20282.61 | 24681.52 | 24722.52 | 25718.21 | 25759.21 |
| Explanatory similarity | 36 | 20215.06 | 20251.06 | 24655.78 | 24691.78 | 25691.73 | 25727.73 |

Note: *partial with profile 5 rank-order correlation freely estimated

To conclude, the results indicated that first, the measurement model was adequately invariant across time and sample and second, the repeated measures latent change profiles were structurally similar in both samples (showed similar mean patterns) as well as explanatory similar in terms of being able to explain similar patterns across the within-class latent changes in socio-emotional competencies.

## **References**

Asparouhov, T., & Muthen, B. (2012). Using Mplus TECH11 and TECH14 to test the number of latent classes. *Mplus web notes* *17*.

Asparouhov, T., & Muthen, B. (2020). Auxiliary Variables in Mixture Modeling: Using the BCH Method in Mplus to Estimate a Distal Outcome Model and an Arbitrary Secondary Model. *Mplus web notes 21*.

Dong, Y., & Peng, C.-Y. J. (2013). Principled missing data methods for researchers. *SpringerPlus*, *2*(1), 222. <https://doi.org/10.1186/2193-1801-2-222>

Ferguson, S. L., G. Moore, E. W., & Hull, D. M. (2020). Finding latent groups in observed data: A primer on latent profile analysis in Mplus for applied researchers. *International Journal of Behavioral Development*, 44(5), 458–468. doi.org/10.1177/0165025419881721

Hallquist, M. N. & Wiley, J. F. (2018). MplusAutomation: An R Package for Facilitating Large-Scale Latent Variable Analyses in Mplus Structural Equation Modeling, 1-18. <https://doi.org/10.1080/10705511.2017.1402334>

Hayes, A. F., & Coutts, J. J. (2020). Use omega rather than Cronbach’s alpha for estimating reliability. But…. *Communication Methods and Measures*, *14*(1), 1-24. <https://doi.org/10.1080/19312458.2020.1718629>

Masyn, K. (2013). *Latent Class Analysis and Finite Mixture Modeling. Oxford Handbook of Quantitative Methods* (pp. 551–611). Oxford University Press.

McArdle, J. J., & Hamagami, F. (2001). *Latent difference score structural models for linear dynamic analyses with incomplete longitudinal data.* In L. M. Collins & A. G. Sayer (Eds.), *Decade of behavior. New methods for the analysis of change* (p. 139–175). American Psychological Association. [https://doi.org/10.1037/10409-005](https://psycnet.apa.org/doi/10.1037/10409-005)

Millsap, R. E., & Yun-Tein, J. (2004). Assessing Factorial Invariance in Ordered-Categorical Measures. *Multivariate Behavioral Research*, *39*(3), 479–515. <https://doi.org/10.1207/S15327906MBR3903_4>

Morin, A. J. S., Meyer, J. P., Creusier, J., & Biétry, F. (2015). Multiple-Group Analysis of Similarity in Latent Profile Solutions: *Organizational Research Methods*. <https://doi.org/10.1177/1094428115621148>

Muthén, L., & Muthén, B. O. (1998-2020). Mplus. User’s guide. Los Angeles, CA: Muthén &

Muthén.

Nylund, K. L., Asparouhov, T., & Muthén, B. O. (2007). Deciding on the number of classes in latent class analysis and growth mixture modeling: A Monte Carlo Simulation Study. *Structural Equation Modeling: A Multidisciplinary Journal, 14(4),* 535–569. <https://doi.org/10.1080/10705510701575396>

Peters, G. Y. (2018, July 18). The alpha and the omega of scale reliability and validity: why and how to abandon Cronbach’s alpha and the route towards more comprehensive assessment of scale quality. <https://doi.org/10.31234/osf.io/h47fv>

Peters, G. J. Y. (2018). _userfriendlyscience: Quantitative analysis made accessible. <https://doi.org/10.17605/osf.io/txequ>

Putnick, D. L., & Bornstein, M. H. (2016). Measurement invariance conventions and reporting: The state of the art and future directions for psychological research. *Developmental Review*, *41*, 71–90. <https://doi.org/10.1016/j.dr.2016.06.004>

Raykov, T., & Marcoulides, G. A. (2004). Using the delta method for approximate interval estimation of parameter functions in SEM. *Structural Equation Modeling, 11*, 621–637. <https://doi.org/10.1207/s15328007sem1104_7>

R Core Team (2020). R: A language and environment for statistical computing. R Foundation for

Statistical Computing, Vienna, Austria. URL: https://www.R-project.org/.

Tein, J.-Y., Coxe, S., & Cham, H. (2013). Statistical Power to Detect the Correct Number of Classes in Latent Profile Analysis. Structural Equation Modeling: A Multidisciplinary Journal, 20(4), 640–657. doi.org/10.1080/10705511.2013.824781

Wickham et al., (2019). Welcome to the tidyverse. *Journal of Open Source Software, 4(43),* 1686, <https://doi.org/10.21105/joss.01686>
